# *Ms*SPL9 Modulates Nodulation under Nitrate Sufficiency Condition in *Medicago sativa*

**DOI:** 10.3390/ijms24119615

**Published:** 2023-06-01

**Authors:** Vida Nasrollahi, Gamalat Allam, Susanne E. Kohalmi, Abdelali Hannoufa

**Affiliations:** 1 Agriculture and Agri-Food Canada, 1391 Sandford Street, London, ON N5V 4T3, Canada; vnasroll@uwo.ca (V.N.); gamalat.allam@agr.gc.ca (G.A.); 2 Department of Biology, University of Western Ontario, 1151 Richmond Street, London, ON N6A 3K7, Canada; skohalmi@uwo.ca

**Keywords:** *Medicago sativa*, *SPL*, *SUNN*, nodulation, nitrate, miRNA

## Abstract

Nodulation in *Leguminous* spp. is induced by common environmental cues, such as low nitrogen availability conditions, in the presence of the specific *Rhizobium* spp. in the rhizosphere. *Medicago sativa* (alfalfa) is an important nitrogen-fixing forage crop that is widely cultivated around the world and relied upon as a staple source of forage in livestock feed. Although alfalfa’s relationship with these bacteria is one of the most efficient between rhizobia and legume plants, breeding for nitrogen-related traits in this crop has received little attention. In this report, we investigate the role of Squamosa-Promoter Binding Protein-Like 9 (SPL9), a target of miR156, in nodulation in alfalfa. Transgenic alfalfa plants with *SPL9*-silenced (*SPL9*-RNAi) and overexpressed (*35S::SPL9*) were compared to wild-type (WT) alfalfa for phenotypic changes in nodulation in the presence and absence of nitrogen. Phenotypic analyses showed that silencing of *MsSPL9* in alfalfa caused an increase in the number of nodules. Moreover, the characterization of phenotypic and molecular parameters revealed that *MsSPL9* regulates nodulation under a high concentration of nitrate (10 mM KNO_3_) by regulating the transcription levels of the nitrate-responsive genes *Nitrate Reductase1* (*NR1*), *NR2*, *Nitrate transporter 2.5* (*NRT2.5*), and a shoot-controlled autoregulation of nodulation (AON) gene, *Super numeric nodules* (*SUNN*). While *MsSPL9–*overexpressing transgenic plants have dramatically increased transcript levels of *SUNN*, *NR1*, *NR2*, and *NRT2.5*, reducing *MsSPL9* caused downregulation of these genes and displayed a nitrogen-starved phenotype, as downregulation of the *MsSPL9* transcript levels caused a nitrate-tolerant nodulation phenotype. Taken together, our results suggest that *MsSPL9* regulates nodulation in alfalfa in response to nitrate.

## 1. Introduction

Unlike animals, the vast majority of plants have to acquire nitrogen, usually in the form of nitrates and ammonium, from the soil. Although nitrogen gas (N_2_) is plentiful in the atmosphere, the biologically active forms of nitrogen are often so limited that they can constrain plant growth. For nodule-forming plants, however, the limitation of nitrogen fixation can be overcome to some extent by acquiring nitrogen from the rhizosphere [1]. While some species-specific factors may be involved, in general, the development of nitrogen-fixing root nodules is controlled by two parallel processes that are initiated by the host plant: first, nodule organogenesis, which is formed from the reinitiation of cell division in the root cortex [1,2], and second, rhizobia infecting the inside of the root hair cells that curl around rhizobia to entrap bacteria, which eventually grow and form infection threads (ITs) [1]. ITs are plant-derived conduits that are capable of crossing cell boundaries to direct rhizobia into the root cortex targets, the site of developing primordia [2,3]. Finally, the rhizobia are released from the ITs into the inner cells in the nodule while remaining encapsulated within a plant membrane. In these organelle-like structures, called symbiosomes, rhizobia are responsible for the reduction of atmospheric di-nitrogen to ammonia by expressing the nitrogenase enzyme [4].

The symbiotic nitrogen fixation of legumes takes place in nodules [2], and signal exchanges for bacterial entry to the nodules take place between host plants and rhizobia [5]. Plant roots release phenolic compounds which attract bacteria to the rhizosphere and subsequently stimulate the secretion of lipo-chito-oligosaccharides, known as nod factors (NF) [1,5]. Recognition of NFs by receptor-like kinases such as nod factor perception (NFP) leads to the induction of a signaling pathway that activates a leucine-rich repeats receptor-like kinase, known as Does not Make Infections2 (DMI2) in *Medicago truncatula* [6]. Secondary signals initiate calcium oscillation in the nuclear region, a process known as calcium spiking [7]. Activation of this signaling pathway requires three components of the nuclear pore—NUP85, NUP133, and NENA [8,9,10]—and the cation channels located on the nuclear envelope, encoded by a single inner-membrane-localized channel, DMI1 [11,12]. Perception of the calcium spiking signature is decoded by a nuclear calcium/calmodulin-dependent protein kinase, DMI3. DMI3 interacts with and subsequently phosphorylates the Interacting Protein of DMI3 (IPD3) [13,14]. DMI3 interacts with the nuclear protein IPD3 and other downstream components, such as two GRAS family proteins—Nodulation Signaling Pathway1 (NSP1) and NSP2—to activate expression of Nodule Inception (NIN), its downstream genes that encode Nuclear Factor YA1 (NF-YA1)/YA2, and ERF Required for Nodulation (ERN2), which are essential for rhizobium infection and nodule organogenesis [15,16,17,18,19,20].

Forming and maintaining nodules is an energy-demanding process, and consequently, excessive nodulation (super-nodulation) can negatively affect plant growth and development [21]. The host plant, therefore, tightly regulates the total root nodule number depending on the metabolic status of the shoot (carbon source) and root (nitrogen source) [22]. To that end, legumes have evolved a negative regulatory pathway called autoregulation of nodulation (AON) that functions systemically through the shoot to maintain an optimal number of nodules [23,24,25]. The nitrogen regulation pathway is activated in root cortical cells during rhizobial infection and nodule development to inhibit nodulation under nitrogen-rich conditions, helping the plant to conserve energy resources [25,26]. Following the initial rhizobial infection events, root-derived nodulation-specific Clavata3/Embryo surrounding region (CLE) peptides, including CLE12 and CLE13 in *M. truncatula* [27], CLE Root Signal1 (CLE-RS1) and CLE-RS2 in *Lotus japonicus*, or Rizobia-induced CLE1 (RIC1) and RIC2 in soybean (*Glycine max*) [28,29], are triggered to activate AON. Following processing, these small functional CLE peptides translocate from the root to the shoot through the xylem [30], where they bind to a specific homodimeric or heterodimeric receptor complex that includes Hypernodulation Aberrant Rant Root Formation1 (HAR1) in *L. japonicus* [30,31,32], *SUNN* in *M. truncatula* [33], or Nodule Autoregulation Receptor Kinase (NARK) in soybean [34]. In *L. japonicus*, *Lj*CLE-RS2 binds to *Lj*HAR1, and the application of *Lj*CLE-RS2 peptide through the xylem was found to inhibit nodulation in wild-type but not in *har1* mutants, showing that the *Lj*HAR1 receptor kinase is required for regulating the AON pathway through the *Lj*CLE peptide [30].

Nodules are induced by common environmental cues such as low nitrogen availability conditions in the presence of the specific *Rhizobium* spp. in the rhizosphere [25]. In legumes, nitrogen is utilized through assimilation regardless of whether it enters the plant as nitrate and ammonium from soil or by fixation of atmospheric nitrogen [35]. Nitrate is absorbed by the root from the external environment using two nitrate transporters, Nitrate Transporter1 (NRT1) and NRT2, which function as low-affinity and high-affinity nitrate transporters, respectively [36]. The nitrate imported into the cells is sequentially reduced into nitrite by Nitrate Reductase (NR) and into ammonium by Nitrite Reductase (NiR) [37]. Ammonium is assimilated into amino acids through the glutamine synthase (GS) and glutamine oxoglutarate aminotransferase (GOGAT) cycle [38]. High levels of nitrogen in soil reduce nodulation and inhibit nitrogen fixation in mature nodules after the addition of nitrogen fertilizers [39]. This regulation of nodulation by nitrate is a part of the AON signaling pathway [40,41]. In *M. truncatula*, Lagunas et al. [42] showed that the AON signaling pathway regulates nitrogen uptake and metabolism even when plants are not nodulating, suggesting that SUNN is involved in controlling nitrogen mobilization even in the absence of rhizobia.

The role of miRNAs in nitrate-regulated root architecture has been reported in the literature. For example, miR167 and its target Auxin Response Factor 8 (ARF8) control lateral root growth in response to nitrate in *Arabidopsis* [43,44]. In addition, miR172 positively regulates nodulation in legumes, as shown in soybean, where overexpression of miR172 resulted in plants with increased nodule number and nitrogen fixation [45]. Nova-Franco et al. [46] also showed similar results in the common bean (*Phaseolus vulgaris*). The miR2111/TML module is also involved in regulating nodulation in legumes, as overexpression of miR2111 or mutations in *TML* caused hypernodulation in *L. japonicus* [47]. In soybean, Yan et al. [48] showed that miR2606b and miR4416 affect nodulation, whereby miR2606b overexpression in roots increased nodule numbers, but overexpression of miR4416 decreased them [48]. Furthermore, De Luis et al. [49] reported that miR171 is involved in the early stages of nodulation (i.e., bacterial infection) in *L. japonicus*. When the expression of miR171 was examined in wild type (WT) and mutant *spontaneous nodule formation1* (*snf1*) and *snf2 L. japonicus* plants, it showed an increase in *Mesorhizobium loti*-inoculated *snf1* and *snf2* mutants compared to WT [49]. It was also found that miR171 targets the *NSP2,* which is an important transcription factor in the nodulation signaling pathway in *M. truncatula* [50]. In addition, Yan et al. [51] showed that miR393 negatively regulates nodule formation in soybean by targeting *Early nodulin 93* (*ENOD93*). Moreover, overexpression of miR319 in *M. truncatula* and common bean resulted in a reduction in the number of nodules in transgenic roots [52,53].

Our previous study found that overexpression of miR156 increased the number of root nodules in alfalfa [54]. Most recently, we showed that miR156-targeted *MsSPL12* has a negative effect on nodulation, as down-regulation of *MsSPL12* increased the number of nodules and nitrogen fixation in alfalfa [55]. Furthermore, it has been shown that there is a regulatory relationship between *MsSPL12* and *CLE13* and that *Ms*SPL12 is involved in the AON symbiotic process in alfalfa [55]. Additionally, Yun et al. [56] reported that the miR156-SPL9 regulatory system in soybean acts as an upstream master regulator of nodulation by targeting and regulating the transcript levels of nodulation genes in this plant. *Gm*SPL9 is a positive regulator of soybean nodulation which directly targets the nodulation master regulator gene, *GmNINa*, and the nodulation marker gene, *GmENOD40*, during nodule formation and development [56]. *Ms*SPL9 in alfalfa has been investigated for its role in drought response in alfalfa, where its downregulation led to improved tolerance to this stress [57]. These findings highlight the role that the miR156/SPL regulatory system plays in nodulation in legume plants.

In the current study, we conducted an investigation into the role of *Ms*SPL9 in nodulation in alfalfa using overexpression (OE) (*35S::SPL9*) and RNAi-silenced *SPL9* (RNAi-*SPL9*) alfalfa plants. We also investigated the role of *Ms*SPL9 in nodulation in response to high concentrations of nitrate.

## 2. Results

### 2.1. Silencing of MsSPL9 Enhances Nodulation

**Figure 1:**
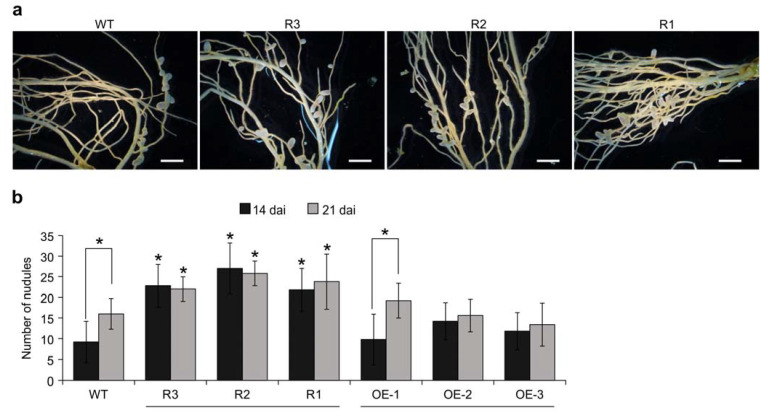
The effect of *MsSPL9* silencing on nodulation. (**a**) Nodule phenotypes of WT and the *SPL9*-RNAi genotypes at 14 dai. Scale bar, 1 mm. (**b**) The number of nodules in WT, *SPL9*-RNAi, and *35S::SPL9* at 14 dai and 21 dai (*n* = 10–14). * Indicates significant differences within time points (14 dai and 21 dai) between WT and *SPL9*-RNAi or *35S::SPL9* plants, and bars indicate significant differences between the two time points using Student’s *t*-test *p* < 0.05. Error bars indicate standard deviation.

Overexpression of miR156 was reported earlier to increase root length and enhance nodulation in alfalfa [54]. As *MsSPL9* was one of the genes that are targeted for silencing by miR156 in this plant, we decided to investigate whether root-related traits are regulated by miR156 through *MsSPL*9 silencing. For that purpose, we studied the root phenotypes in WT, *SPL9*-RNAi (R1, R2, and R3), and *35S::SPL9* (OE-1, OE-2, and OE-3) genotypes (Figure 1). To determine the ability of *SPL9*-RNAi and *35S::SPL9* transgenic plants to form symbiotic nodules, three-week-old (three weeks post-cutting) rooted plants were inoculated with *Sinorhizobium meliloti* for a period of 14 or 21 days after inoculation (dai). At 14 dai, *SPL9*-RNAi plants showed an increase in nodulation of 2.5-, 2.9-, and 2.4-fold in R3, R2, and R1, respectively, compared to WT (Figure 1a,b). The total nodule number also was increased in *SPL9*-RNAi genotypes compared to WT at 21 dai, showing 1.4-, 1.6-, and 1.5-fold in R3, R2, and R1, respectively, compared to WT (Figure 1b). Although no significant differences in nodule numbers were observed between *SPL9*-RNAi genotypes at 14 dai and 21 dai, nodulation was increased in WT at 21 dai compared to 14 dai (Figure 1b).

To determine the ability of *35S::SPL9* transgenic plants to form symbiotic nodules, three weeks after cutting, the rooted transgenic and WT plants were inoculated with *S. meliloti* for 14 and 21 days. No significant differences in nodule numbers were observed between *35S::SPL9* genotypes and WT at 14 dai nor 21 dai (Figure 1b). However, among the *35S::SPL9* genotypes, the total nodule number was significantly increased in OE-1 at 21 dai compared with this genotype at 14 dai (Figure 1b).

These results suggest that the transcript levels of *MsSPL9* are negatively correlated to nodulation in alfalfa.

### 2.2. MsSPL9 Silencing Affects Nodulation-Related Genes

**Figure 2:**
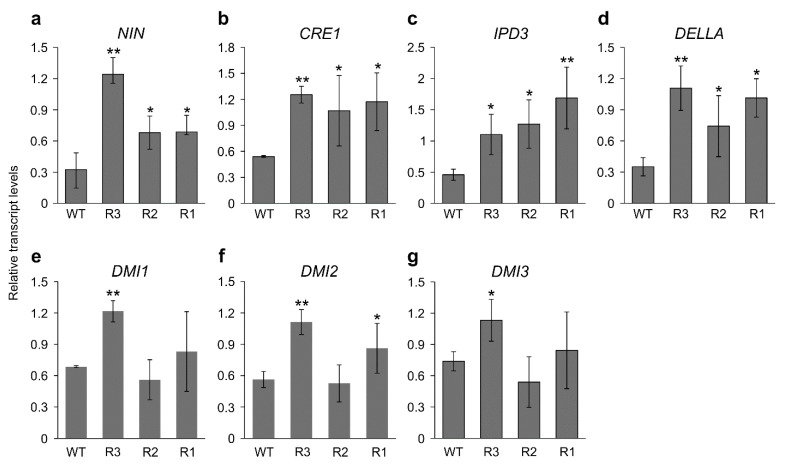
Transcript analysis of nodulation-related genes in *SPL9*-RNAi genotypes. Relative transcript levels of (**a**) *NIN,* (**b**) *CRE1*, (**c**) *IPD3*, (**d**) *DELLA*, (**e**) *DMI1*, (**f**) *DMI2*, and (**g**) *DMI3* in WT and *SPL9*-RNAi roots at 14 dai. Transcript levels are shown relative to WT after being normalized to ADF1 and *elF4A* reference genes. *, ** Indicate significant differences relative to WT using Student’s *t*-test (*n* = 3) *p* < 0.05, *p* < 0.01, respectively. Error bar indicates standard deviation.

Given the above finding that *SPL9*-RNAi alfalfa plants have enhanced nodulation in the symbiotic relationship with *S. meliloti*, we examined transcript levels of several nodulation-related genes at 14 dai in inoculated roots of alfalfa (Figure 2). These genes include *NIN* [17], *CRE1* [58], *IPD3* [13], *DELLA* [59], *DMI1* [11], *DMI2* [6], and *DMI3* [13].

Of the tested genes, *NIN*, *CRE1*, *IPD3* and *DELLA* were significantly upregulated in all the *SPL9*-RNAi genotypes (R1, R2 and R3) at 14 dai (Figure 2a–d). While *DMI1* and *DMI3* were upregulated in only one of the *SPL9*-RNAi genotypes (R3) (Figure 2e,g), significant effects of *MsSPL9* silencing on *DMI2* transcript levels were observed in roots of two *SPL9*-RNAi genotypes, R3 and R1, at 14 dai (Figure 2f). These findings suggest the involvement of *Ms*SPL9 in nodulation in alfalfa-*S. meliloti* symbiosis.

### 2.3. MsSPL9 Silencing Attenuates the Effect of Nitrate on Nodulation

**Figure 3:**
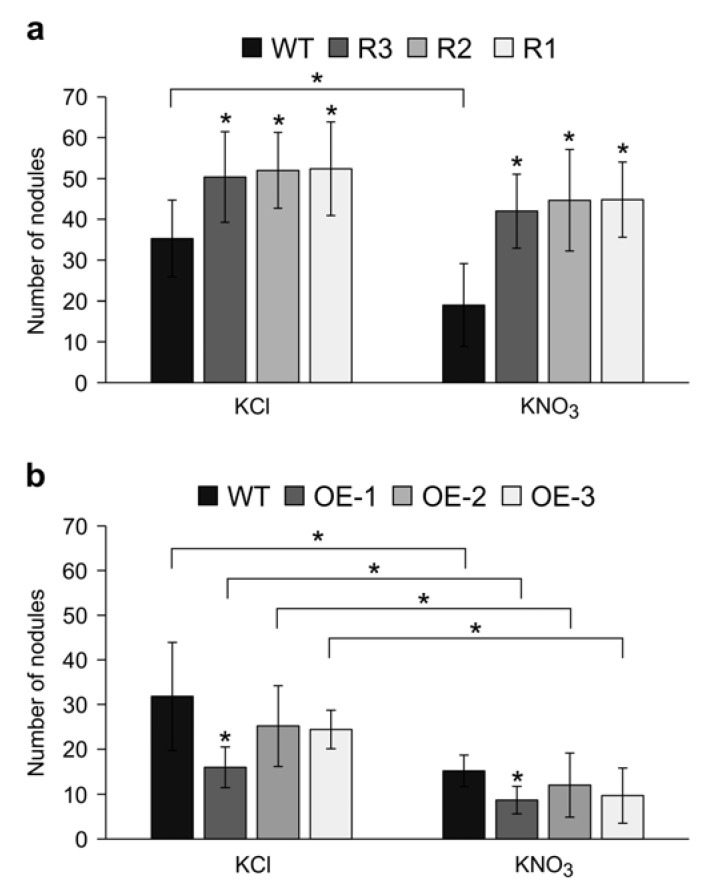
Effect of 10 mM KNO_3_ on nodulation phenotype in *SPL9*-RNAi and *35S::SPL9* genotypes. The average numbers of nodules at 21 dai in (**a**) WT and *SPL9*-RNAi, and (**b**) WT and *35S::SPL9* genotypes (*n* = 10–14). * Indicates significant differences within conditions between WT and *SPL9*-RNAi or *35S::SPL9* plants, and bars indicate significant differences between conditions using Student’s *t*-test *p* < 0.05. Error bars indicate standard deviation.

Nitrogen abundance in the soil inhibits nodulation, and this regulatory process is a part of the AON pathway [41,60]. Given the effects of *Ms*SPL9 on nodulation, we assessed whether the nodulation capacity of *S. meliloti-*inoculated *SPL9*-RNAi transgenic plants, R1, R2, and R3, was affected by nitrate treatment. The number of nodules was compared between WT and *SPL9*-RNAi plants treated with 10 mM KNO_3_ or KCl at 21 dai. Under watering with 10 mM KCl, *SPL9*-RNAi transgenic plants produced significantly more nodules compared to WT. When watered with 10 mM KNO_3_, WT plants showed a reduction in the nodule number compared to when treated with KCl (Figure 3a), but transgenic *SPL9*-RNAi plants maintained nodulation under both treatments. In fact, nodule numbers were not noticeably affected by KNO_3_ treatment in these transgenic plants (Figure 3a). These results indicate that silencing of *MsSPL9* prevents nitrate inhibition of nodulation in alfalfa.

To further investigate the role of *Ms*SPL9 in nodulation under nitrate treatment, we analyzed the nodulation phenotype of transgenic alfalfa plants overexpressing *MsSPL9* in the presence of either 10 mM KCl or KNO_3_ (Figure 3b). The number of nodules was only significantly decreased in one of the *35S::SPL9* transgenic plants, OE-1, which expresses the highest level of *MsSPL9* compared to WT, in both conditions (watering with KCl or KNO_3_) (Figure 3b). In fact, there was no significant difference in the nodule numbers of OE-2 and OE-3 compared to WT. However, when plants were watered with 10 mM KNO_3_, WT and all *35S::SPL9* transgenic plants produced significantly fewer nodules than plants watered with 10 mM KCl (Figure 3b). This result suggests that overexpression of *MsSPL9* causes hypersensitivity to nitrate inhibition of nodulation in alfalfa.

### 2.4. Differential Gene Expression in SPL9-RNAi and 35S::SPL9 Genotypes

**Figure 4:**
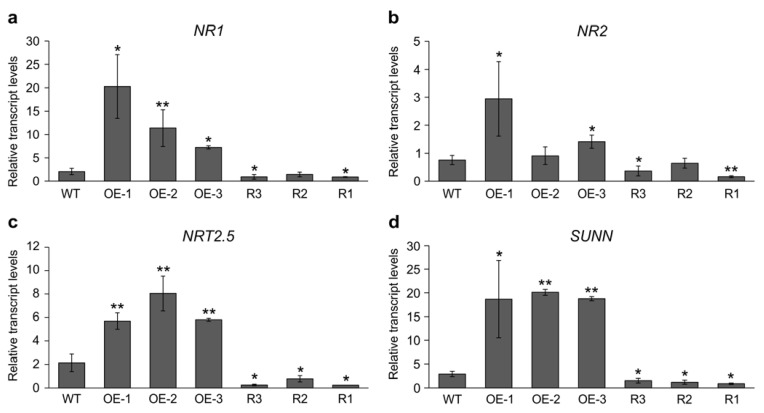
Transcript analysis of nitrate signaling pathway genes in *SPL9*-RNAi and *35S::SPL9* genotypes. Relative transcript levels of (**a**) *NR1*, (**b**) *NR2*, (**c**) *NRT2.5*, and (**d**) *SUNN* in *SPL9*-RNAi and *35S::SPL9* at 14 dai. Transcript levels are shown relative to WT after being normalized to ADF1 and *elF4A* reference genes. *, ** Indicate significant differences relative to WT using Student’s *t*-test (*n* = 3) *p* < 0.05, *p* < 0.01, respectively. Error bar indicates standard deviation.

To shed light on the molecular events associated with *Ms*SPL9 function in nodulation in response to nitrate, we investigated the transcript levels of three nitrate-responsive genes: *NR1*, *NR2*, and a high-affinity *NRT2.5* in inoculated roots of alfalfa plants with altered expression of *MsSPL9* (*SPL9*-RNAi and *35S::SPL9*) and WT. Overexpression of *MsSPL9* resulted in higher transcript levels of *NR1*, *NR2*, and *NRT2.5*, except for OE-2, which showed no significant changes for *NR2* compared to WT (Figure 4a–c).

By contrast, reduced *MsSPL9* in the *SPL9*-RNAi transgenic plants caused lower transcript levels of *NR1* and *NR2* (Figure 4a,b), except for one *SPL9*-RNAi plant, R2, that did not show any significant change for these two genes compared to WT. *NRT2.5* transcript levels were lower in all *SPL9*-RNAi plants (Figure 4c). We also analyzed the transcript levels of a shoot-controlled AON gene, *SUNN,* in *SPL9*-RNAi transgenic plants. In *M. truncatula*, *SUNN* is involved in control of nitrogen mobilization, as control plants appear to be able to transport more nitrogen to the shoot, compared with *sunn-1* loss of function mutant plants [42]. Our results showed that silencing *MsSPL9* was accompanied by lower transcript levels of *SUNN* (Figure 4d). We also detected the expression of this AON gene in *35S::SPL9*. Expression levels of *SUNN* were markedly higher in the *MsSPL9* overexpressing transgenic plants than in WT (Figure 4d). These results are consistent with the increased number of nodules in *SPL9*-RNAi compared to those of *35S::SPL9* and WT. Since SUNN is an important component in the AON signaling pathway, these results suggest that *Ms*SPL9 is involved in regulating AON in alfalfa.

### 2.5. Root Development Is Balanced Differently in WT and SPL9-RNAi

**Figure 5:**
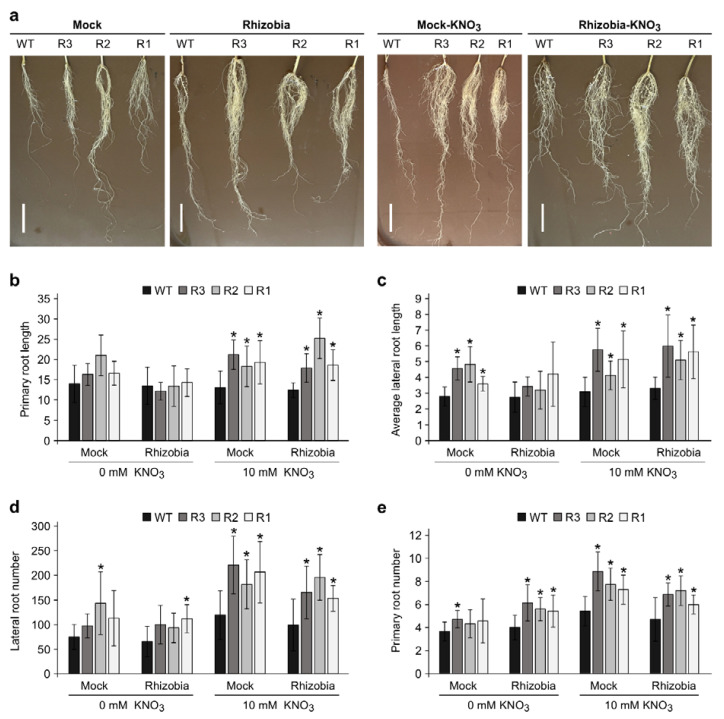
WT and *SPL9*-RNAi root system architecture changes after KNO_3_ treatment, with effects dependent on rhizobia-inoculation status. (**a**) Root architecture phenotype of inoculated WT and the *SPL9*-RNAi genotypes with *S. meliloti* at 21 dai growing in nitrate-starved substrate or watered with 10 mM KNO_3_. Scale bar, 4 cm. (**b**) Primary root length, (**c**) average lateral root length, (**d**) numbers of the lateral root, and (**e**) numbers of the primary root. * Indicates significant differences relative to WT using Student’s *t*-test (*n* = 10) *p* < 0.05. Error bar indicates standard deviation.

To gain an insight into the function of *Ms*SPL9 in root architecture and nodulation in response to nitrate, we analyzed root system architecture (RSA) in *SPL9*-RNAi and WT alfalfa plants. The transgenic and WT plants were grown in the absence of nitrate and treated with either *S. meliloti* (“rhizobia”) or mock for 14 days. The plants were then treated with either 10 mM KNO_3_ or sterile water for 14 more days to study the individual and combinatorial effects of these treatments on RSA. We found that between the four conditions (Mock, Rhizobia, Mock-KNO_3_, and Rhizobia-KNO_3_), there was no significant difference in primary root (PR) and lateral root (LR) length in WT plants (Figure 5a and Figure S1a,b), although these traits were significantly different in *SPL9*-RNAi plants in response to nitrate and inoculation with rhizobia (Figure 5a and Figure S1a,b).

In the presence of KNO_3_, *SPL9*-RNAi plants had significantly longer PR than WT, either with or without rhizobia inoculation (Figure 5b), while all plants had similar PR lengths in the absence of nitrate. Inoculated *SPL9-*RNAi did not show any significant change in LR length compared to WT in the absence of nitrate, but they showed longer LR compared to WT in the other three conditions (Figure 5c). In addition, LRs were shorter in inoculated R2 and R3 in the absence of KNO_3_ than when it was present (Figure S1b).

When *SPL9*-RNAi plants were treated with KNO_3_, they showed significantly more lateral roots than WT regardless of inoculation status (Figure 5d). In the absence of KNO_3_, all plants had similar LR numbers, except for R2 in mock and R1 in rhizobia-inoculated conditions that showed an increase in the number of LRs compared to WT (Figure 5d). Furthermore, the number of PRs was significantly higher in *SPL9*-RNAi plants in all the conditions, except for mock plants growing in the absence of KNO_3_; in fact, only R3 from this group showed an increase in the number of PRs compared to those for WT (Figure 5e).

These results suggest the involvement of *Ms*SPL9 in the regulation of root architecture in response to nitrate and inoculation with rhizobia.

### 2.6. Plant Biomass in SPL9-RNAi under KNO_3_ Treatment Are Affected by Inoculation Status

**Figure 6:**
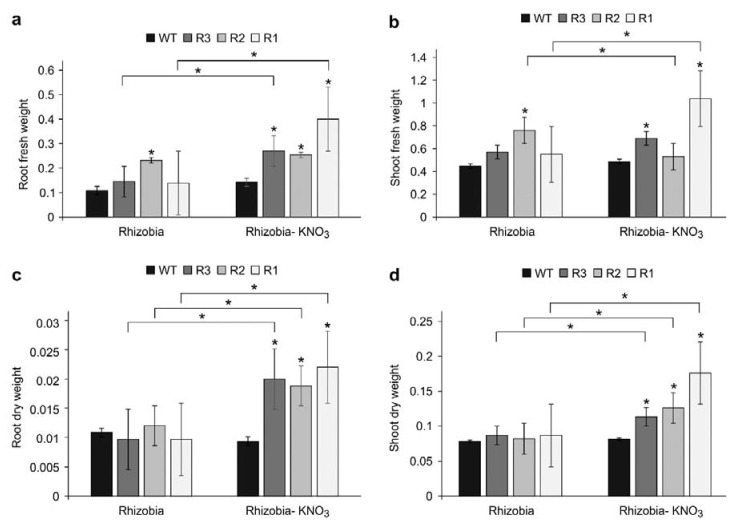
WT and *SPL9*-RNAi root and shoot weight changes after KNO_3_ treatment. (**a**) Root fresh weight (RFW), (**b**) shoot fresh weight (SFW), (**c**) root dry weight (RDW), and (**d**) shoot dry weight (SDW). * Indicates significant differences within conditions between WT and *SPL9*-RNAi plants, and bars indicate significant differences between conditions using Student’s *t*-test (*n* = 10) *p* < 0.05. Error bar indicates standard deviation.

Based on the differential expression of some nitrate signaling genes in the *SPL9-*RNAi plants (Figure 4), we decided to determine whether this factor also had an effect on whole plant biomass. We measured the root and shoot fresh and dry weight of WT and *SPL9*-RNAi plants grown in vermiculite. We inoculated plants with *S. meliloti*, and 14 days later, the plants were treated with either 10 mM KNO_3_ or sterile water for 14 more days to study the individual and combinatorial effects of treatments on root and shoot biomass. Increased root fresh weight was observed in KNO_3_-treated *SPL9*-RNAi plants in addition to the untreated R2 plants compared to WT in each treatment (Figure 6a).

Differences in root fresh weight were not observed between treatments in WT and R2, while R1 and R3 root fresh biomass were increased in response to KNO_3_ compared to untreated plants (Figure 6a). WT and transgenic plants were also distinguishable between and within conditions when examining shoot fresh biomass. In the absence of KNO_3_, there were no significant changes between *SPL9*-RNAi plants and WT in shoot fresh weight, except for R2, which showed an increase (Figure 6b). In the presence of KNO_3_, two of the *SPL9*-RNAi plants, R1 and R3, showed an increase in shoot fresh weight compared to WT. When comparing the shoot fresh weight between the treatments, only R2 and R3 showed an increase in response to KNO_3_ (Figure 6b).

We also measured root and shoot dry biomass in the KNO_3_-treated and untreated plants. In the absence of KNO_3_, WT and *SPL9*-RNAi plants were indistinguishable when examining root and shoot dry biomass (Figure 6c,d). However, in the presence of KNO_3_, root and shoot dry weight were significantly increased in all the *SPL9*-RNAi plants compared to WT (Figure 6c,d). When comparing the root and shoot dry biomass between the two conditions (KNO_3_-treated and untreated plants), only WT did not show any changes, while the dry biomass was significantly increased in *SPL9*-RNAi plants in response to KNO_3_ (Figure 6c,d).

## 3. Discussion

The role of miR156 in plant growth and development has been well documented [61,62,63]. Previous research has shown that overexpression of *miR156* in alfalfa (miR156-OE) resulted in increased nodulation, improved nitrogen fixation, and enhanced root regenerative capacity during vegetative propagation [64]. Whereas the role of the miR156-regulated *MsSPL9* in drought stress response has been well characterized in alfalfa [57], no studies have been conducted on the possible role of miR156/SPL9 module in nodule development in alfalfa. In the present study, we analyzed transgenic alfalfa plants with altered transcript levels of *MsSPL9,* including *SPL9*-RNAi and *35S::SPL9,* to investigate the role of *Ms*SPL9 in root architecture.

As *MsSPL9* is one of the genes regulated by miR156 in alfalfa, we decided to investigate its involvement in nodulation. Formation of root nodules in association with rhizobia in leguminous plants is a complex process that governs the mutually beneficial relationship between the plants and their compatible rhizobia and includes the downstream components of signaling pathways that trigger changes in gene expression in both partners [1,65,66]. The signals that provide bacterial access to the plant, leading eventually to nodule organogenesis, have been well studied in legume species [65,66]. miR156/SPL was shown to play a role in nodulation in legume plants, including alfalfa, where overexpression of miR156 increased the number of root nodules [54]. Most recently, we showed that miR156-targeted *MsSPL12* and its downstream target *AGL6* are involved in the regulation of nodulation in this plant [55]. In fact, *Ms*SPL12 plays a negative role in nodulation in alfalfa, as its down-regulation was concomitant with changes in gene expression in both partners, alfalfa roots and *S. meliloti* [55], resulting in enhanced nodulation.

In the current study, we showed that *Ms*SPL9 has a negative effect on nodulation in alfalfa. While overexpression of *MsSPL9* resulted in no evident change in nodulation, silencing of this gene (*SPL9*-RNAi) increased nodulation in these plants. Based on these results, miR156/SPL was shown to play a role in nodulation in legume plants. However, the role of miR156/SPL9 in nodulation may be species-specific, as an increase in nodulation was also reported in the other study for *GmSPL9* overexpression in soybean plants. Yun et al. [56] reported that *Gm*SPL9 in soybean, which is phylogenetically close to *Ms*SPL9 in alfalfa [67], acts as an upstream positive regulator of nodulation by targeting and regulating the expression of nodulation genes. As such, overexpression of *GmSPL9* in soybean reduced nodule numbers [56]. *Gm*SPL9 regulates nodulation in soybean by directly binding to the promoter of miR172c and activating its expression [56]. *Gm*SPL9 also directly targets *GmNINa* and *GmENOD40*, which are the nodulation master regulators and nodulation marker genes, respectively, during nodule formation and development [56]. In addition to the potential species specificity of SPL9 function in nodulation, the discrepancy could be explained by the dose-dependent effects of SPLs. Hanly et al. [57] showed that only two of the *SPL9*-RNAi alfalfa plants (R2 and R3), the genotypes with the lowest *MsSPL9* transcript abundance, improved tolerance to drought. Similar findings were reported by Feyissa et al. [68] for *MsSPL13*, where only *SPL13*-RNAi alfalfa plants with decreased *MsSPL13* transcript levels but over a certain threshold showed significant drought tolerance.

Intriguingly, we also found that *Ms*SPL9 regulates the transcription levels of a shoot-controlled AON gene, *SUNN,* in alfalfa. We found that overexpression and silencing of *MsSPL9* resulted in up- and down–regulation of *SUNN*, respectively (Figure 4d). These results suggest a link between the increased and decreased number of nodules in *SPL9*-RNAi and *35S::SPL9*, respectively, compared to WT, and the expression level of *SUNN* in these plants. Moreover, because SUNN is an important component in the AON signaling pathway, these results also suggest that *Ms*SPL9 is involved in regulating the AON pathway in alfalfa and that *Ms*SPL9 may function upstream of SUNN to regulate alfalfa nodulation. It is noteworthy that *NIN*, *CRE1*, *IPD3,* and *DELLA* transcript levels were significantly higher in all the *SPL9*-RNAi genotypes, which supports the hypothesis of their possible regulation by *Ms*SPL9 acting as an essential regulator of nodule organogenesis in alfalfa. However, further experiments are required to identify the downstream targets of *Ms*SPL9 in roots.

Legume plants can fix atmospheric N_2_ into ammonia and assimilate inorganic nitrogen sources due to the symbiotic relationship between rhizobia and root nodules. However, to conserve energy, plants inhibit nodulation under conditions of nitrate abundance in the rhizosphere, resulting in decreased nodule number, nodule mass, nitrogenase activity, and accelerated nodule senescence [60]. Repressing nodulation, sufficient nitrogen status is part of the AON signaling pathway [40,41]. As the *SPL9*-RNAi plants showed an increase in nodulation, we tested the relationship between nitrate and the miR156/SPL9 regulatory system. We found that while silencing *MsSPL9* has enhanced tolerance to nitrate during nodulation, overexpression of *MsSPL9* causes hypersensitivity to nitrate inhibition of nodulation. In fact, under nitrate-sufficient conditions, nodule numbers were not noticeably affected by nitrate treatment in rhizobia-inoculated roots of *SPL9*-RNAi plants. On the other hand, WT and all *35S::SPL9* transgenic plants produced significantly fewer nodules under nitrate-sufficient conditions than plants grown under control conditions. In our previous work, we showed that *SPL12*-RNAi plants developed more active nodules relative to WT under nitrate-sufficient conditions, demonstrating the role of miR156/SPL12 modules in controlling symbiotic nodulation in alfalfa [69]. Given that *Ms*SPL9 and *Ms*SPL12 affect nodulation under nitrate treatment during nodule development [69], it is conceivable that miR156 dynamically controls the process of nodulation by regulating *Ms*SPL transcription factors in alfalfa. The observations that nodulation was regulated by miR156 overexpression in alfalfa [54], *L. japonicus* [62], and soybean [56] support the notion that the central regulatory role of *miR156-*SPL modules on symbiotic nodulation may be conserved in leguminous plants, such as alfalfa, *L. japonicus*, and soybean. In *Arabidopsis*, *At*SPL*9* acts as a potential nitrate regulatory hub and *AtSPL9* expression is affected by nitrate. In addition, the transcript levels of *AtNRT1*.*1*, *AtNIA2*, and *AtNiR* significantly increased in response to nitrate in *AtSPL9* overexpressed *Arabidopsis* plants [70]. Furthermore, miR172c was shown to act as a signaling component of the nitrate-dependent AON in common bean, where it decreases the sensitivity of nodulation inhibition by nitrate [46]. Common bean plants overexpressing miR172 showed an increase in active nodules in the presence of nitrate [46]. In tomato (*Solanum lycopersicum*), it was reported that an *Sl*SPL transcription factor, LeSPL-CNR, directly binds to the promoter of *SlNIA,* resulting in repressing its expression [71]. LeSPL-CNR was further shown to negatively regulate *SlNIA* transcription levels in response to cadmium (cd) stress [71]. In *M. truncatula*, *NIN-like protein 1* (*nlp1*) mutants had dramatically reduced induction of *NiR1* and *NRT2.1* and displayed nitrogen-starved phenotypes, including reduced nodule formation and nitrogen fixation in response to nitrate [40]. In the current study, we showed that *MsSPL9* overexpressing transgenic plants have dramatically reduced transcript levels of the nitrate-responsive genes *NR1*, *NR2*, and *NRT2.5*. By contrast, reduced *MsSPL9* in the *SPL9*-RNAi transgenic plants caused downregulation of *NR1* and *NR2*, and *NRT2.5* and displayed a nitrogen-starved phenotype, as downregulation of the *MsSPL9* expression caused a nitrate-tolerant nodulation phenotype. In fact, nodulation did not decrease in *SPL9*-RNAi plants under nitrate-sufficient conditions.

Moreover, we found *Ms*SPL9 to be involved in the regulation of root architecture in response to nitrate and inoculation with rhizobia. *SPL9*-RNAi plants had increased number and length of PR and LR in response to high concentrations (10 mM) of nitrate. This increase could be explained by the regulation of the *SUNN* gene (an important component in the AON signaling pathway) by *Ms*SPL9, as described earlier (Figure 4d). This gene could be key in the regulation of root architecture in response to the internal and external nitrogen signals to mount an appropriate developmental response. In *M. truncatula*, *sunn-1* mutants had significantly more LRs and greater lateral root density than WT plants when treated with 5 mM NH_4_NO_3_ [42]. In addition, *sunn-1* mutants showed shorter LRs compared to WT plants when inoculated with rhizobia [42]. The *har1* mutant in *L. japonicus*, also had higher LR density and a shorter root system in the absence of rhizobia [72]. Lagunas et al. [42] showed that in *M. truncatula* the AON signaling pathway regulates nitrogen uptake and metabolism, as many nitrogen transporter genes were found to respond to nitrogen in *sunn-1* mutants but were not repressed by nitrogen in WT plants. It has also been shown that the dry weight in *sunn-1* rhizobia-inoculated plants increased more quickly than in WT [42]. In the current study, we showed that root and shoot dry weight were significantly increased in all *SPL9*-RNAi plants compared to WT in the presence of nitrate (Figure 6c,d). Based on our results and the results from previous studies, it could be argued that SUNN is involved in the control of nitrogen mobilization and that *Ms*SPL9 may function upstream of SUNN.

## 4. Materials and Methods

### 4.1. Plant Material and Growth Conditions

*Medicago sativa L.* (alfalfa) clone N4.4.2 [73] was obtained from Daniel Brown (Agriculture and Agri-Food Canada, London, ON, Canada) and was used as a wild-type (WT) genotype. Alfalfa genotypes with reduced expression levels of *MsSPL9*, *SPL9*-*RNAi* (R1, R2, and R3) and plants overexpressing *SPL9*, *35S::SPL9* (OE-1, OE-2, and OE-3) were obtained from our previous study [57]. WT and transgenic alfalfa plants were grown under greenhouse conditions at 21–23 °C, 16-h light/8-h dark per day, a light intensity of 380–450 W/m^2^ (approximately 500 W/m^2^ at high noon time), and a relative humidity of 56% for the duration of all experiments. Because of the obligate outcrossing nature of alfalfa, WT and transgenic alfalfa were propagated by rooted stem cuttings to maintain the genotype throughout the study. Stem-cutting propagation and morphological characterization of alfalfa plants were carried out as described in [54].

### 4.2. Determination of Nodule Numbers

To determine the number of nodules, plants were examined at 14 and 21 days after inoculation (dai) with *Sinorhizobium meliloti* Sm1021. To eliminate potential microbial contamination, equipment was surface-sterilized using 1% sodium hypochlorite, while vermiculite and water were sterilized by autoclaving for 1 h. *S. meliloti* Sm1021 strain was cultured on a Yeast Extract Broth agar [74] for two days at 28 °C. A single colony was then inoculated in liquid TY medium and incubated at 28 °C to an optical density OD_600_ nm of 1.5. The 3-week-old rooted stems were inoculated by applying 5 mL of the bacterial suspension or sterilized water (non-inoculated control) into each pot containing rooted alfalfa stem. The plants were then kept on a bench in the greenhouse and watered with distilled water once a week. Two and three weeks after inoculation with *S. meliloti*, the roots were removed from the soil and the total number of nodules from each stem was counted. At least 10 biological replicates per genotype were used, and the experiment was repeated three times.

### 4.3. Nitrate Treatment

To explore if *Ms*SPL9-related regulation of nodulation is affected by nitrate, the nodulation test was performed upon treatment with this nutrient. WT and *SPL9*-RNAi alfalfa stem cuttings were grown on vermiculite for 21 days and were then inoculated with *S. meliloti* Sm1021 for 14 days. The 14-day-old inoculated transgenic and WT plants were watered with 10 mM potassium nitrate (KNO_3_) or potassium chloride (KCl) twice a week for three weeks. Effects on nodulation were studied by counting the number of nodules. Other phenotypic characterizations, including fresh and dry weight of root and shoot, number of main and lateral roots, length of primary roots, and length of lateral roots on the longest root were also performed and from this the average LR length was calculated. These phenotypic characterizations were performed on nitrate-treated and non-treated WT and *SPL9*-RNAi alfalfa with approximately 10 biological replicates of each genotype. Aboveground tissue was determined by decapitating the plant approximately above the media line, and any tissue below this point was considered roots. Tissue fresh weight (FW) was measured at the time of harvest, and dry weight (DW) was determined after the tissue was baked at 65 °C for 5 days. Root length was considered the length from the top of the root crown to the tip of the longest root. The roots directly emerging from the stem were considered main roots, while those that emerged from the main roots were counted as lateral roots. The entire experiment was repeated twice under the same growth and nitrate treatment conditions to test the reproducibility of the results.

### 4.4. RNA Extraction, Reverse Transcription, and RT-qPCR

Alfalfa roots were collected, flash-frozen in liquid nitrogen, and stored at −80 °C until further use. Approximately 100 mg fresh weight was used for total RNA extraction using Total RNA Purification Kit (Norgen Biotek, Thorold, ON, Canada, Cat #25800) for roots. Tissue was homogenized using a PowerLyzer^®^24 bench top bead-based homogenizer (Cat #13155) according to the manufacturer’s manual. Approximately 500 ng of Turbo DNase (Invitrogen, Waltham, MA, USA, Cat #AM1907) treated RNA was used to generate cDNA using the iScript cDNA synthesis kit (Bio-Rad, Hercules, CA, USA, Cat # 1708891). Transcript levels were analyzed by RT-qPCR using a CFX96 TouchTM Real-Time PCR Detection System (Bio-Rad) and SsoFast™ EvaGreen^®^ Supermixes (Bio-Rad Cat # 1725204) using gene-specific primers. Each reaction consisted of 2 μL of cDNA template, 0.5 μL forward and reverse gene-specific primers (10 μM each) (Table S1), and 5 μL SsoFast Eva green Supermix, then topped up to 10 μL with ddH_2_O. For each sample three or four biological replicates were analyzed, and each biological replicate was tested using three technical replicates. Transcript levels were analyzed relative to two reference genes: *actin–depolymerizing protein 1* (*ADF1*) and *elongation initiation factor 4A* (*elF4A*) (primers are listed in Table S1).

### 4.5. Statistical Analysis

Statistical analyses were performed using Microsoft Excel spreadsheet software. Pairwise comparisons were made using Student’s *t*-test with either equal or unequal variance. The significant differences between sample means for three or more data sets were calculated using the one-way analysis of variance (ANOVA) where appropriate.

## Data Availability

All relevant data can be found within the article and its supporting materials.

## Supplementary Materials

The following supporting information can be downloaded at: https://www.mdpi.com/article/10.3390/ijms24119615/s1.
