# Supplementary material for: MsSPL9 Modulates Nodulation under Nitrate Sufficiency Condition in Medicago sativa
Source: Int J Mol Sci. 2023 Jun 1;24(11):9615. doi: 10.3390/ijms24119615 (PMC10253965; doi:10.3390/ijms24119615)
Supplement: Supplementary file 1 [file ijms-24-09615-s001.zip › Figure S1.pdf]

# *Ms*SPL9 Modulates Nodulation under Nitrate Sufficiency Condition in *Medicago sativa*

**Vida Nasrollahi [1,2], Gamalat Allam [1,2], Susanne E. Kohalmi [2] and Abdelali Hannoufa [1,2,*]**

[1] Agriculture and Agri-Food Canada, 1391 Sandford Street, London, ON N5V 4T3, Canada; vnasroll@uwo.ca (V.N.); gamalat.allam@agr.gc.ca (G.A.)

[2] Department of Biology, University of Western Ontario, 1151 Richmond Street, London, ON N6A 3K7, Canada; skohalmi@uwo.ca

* Correspondence: abdelali.hannoufa@agr.gc.ca

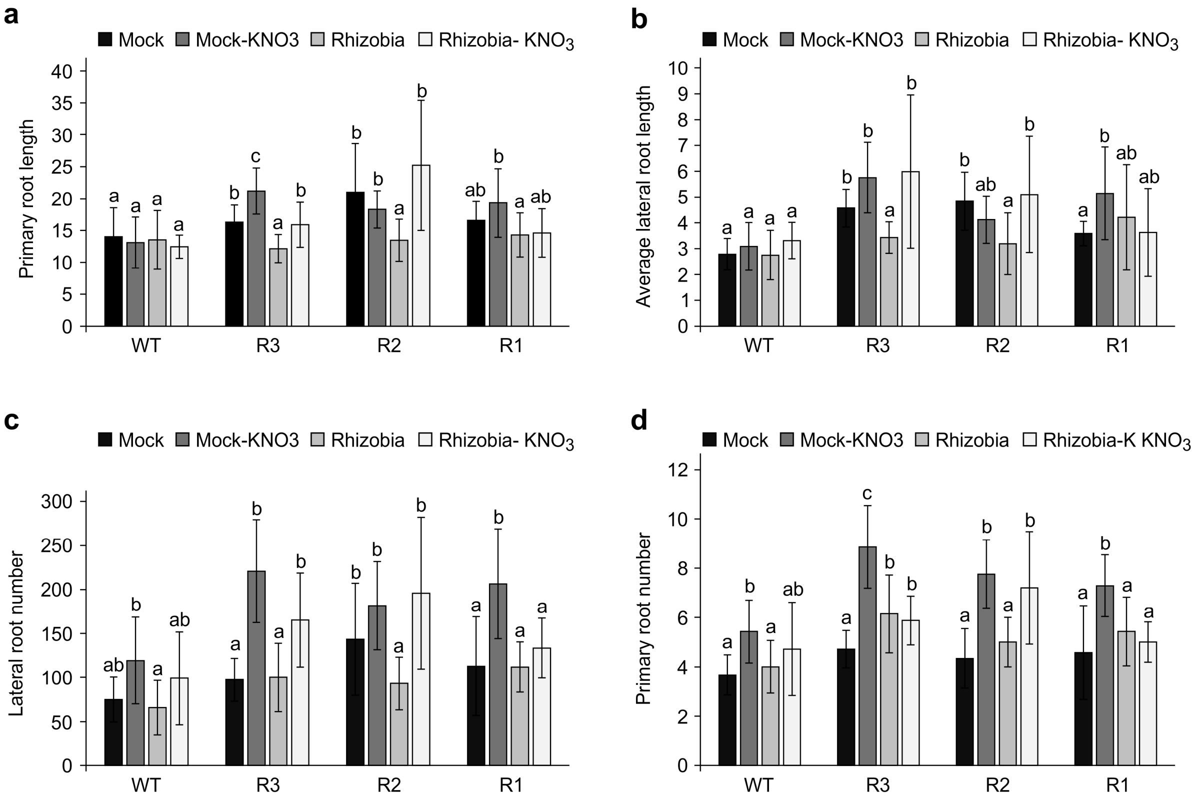


**Figure S1.** WT and *SPL9*-RNAi root system architecture changes after $KNO_3$ treatment, with effects dependent on rhizobia-inoculation status. Root architecture analysis of WT and the *SPL9*-RNAi inoculated with *S. meliloti* or not inoculated genotypes growing in nitrate-starved substrate or watered with 10 mM $KNO_3$. **(a)** Primary root length, **(b)** Average lateral root length, **(c)** Numbers of lateral root, and **(d)** Numbers of primary root, (n = 10). Significant difference from ANOVA was followed by Post hoc Tukey ($P<0.05$) multiple comparisons test indicated with different letters, and have been determined separately for each treatment (Mock, Mock-$KNO_3$, Rhizobia, and Rhizobia-$KNO_3$). Error bars indicate standard deviation.
